# Simulating human behavior under earthquake early warning

**DOI:** 10.1016/j.heliyon.2025.e42060

**Published:** 2025-01-17

**Authors:** Matthew Wood, Sara K. McBride, Xilei Zhao, Dare Baldwin, Elizabeth S. Cochran, Xiaojian Zhang, Nicolas Luco, Ruggiero Lovreglio, Tom Cova

**Affiliations:** a Department of Geography, University of Utah, 260 S Central Dr, RM 4625, Salt Lake City, UT, 84112, USA; b U.S. Geological Survey, Earthquake Science Center, Moffett Field, CA, 94040, USA; c Department of Civil and Coastal Engineering, University of Florida, 1949 Stadium Rd, Gainesville, FL, 32611, USA; d Department of Psychology/Clark Honors College, University of Oregon, Eugene, OR, 97405, USA; e U.S. Geological Survey, Earthquake Science Center, Pasadena, CA, USA; f U.S. Geological Survey, Geologic Hazards Science Center, Golden, CO, 80401, USA; g School of Built Environment, Massey University, Albany, North Shore, Auckland, 0745, New Zealand

**Keywords:** EEW (earthquake early warning), ABM (agent-based modeling), Protective action, Simulation

## Abstract

Earthquakes are a rapid-onset hazard where advance planning and learning plays a key role in mitigating injuries and death to individuals. Recent advances in earthquake detection have resulted in the development of earthquake early warning (EEW) systems. These systems can provide advance warning to predetermined geographic regions that an earthquake is in progress, which may result in individuals receiving warning seconds before significant shaking is felt at their location. This additional time could allow individuals to take more effective protective actions during the immediate disaster. To demonstrate this, we created an agent-based simulation of a basic apartment that allowed us to randomly and repeatedly simulate an individual receiving and responding to an EEW message. The results of our preliminary simulation show that, in our study environment, earthquake early warning alerts have the potential to allow for sufficient time for individuals to take protective actions.

## 1 Introduction

Although several advancements have been made in disaster predictions, earthquakes are still particularly difficult to mitigate, as it is not possible to predict the exact timing, place, depth, and magnitude of an earthquake [1]. That said, it is possible to identify faults and areas where enough elastic strain has accumulated to produce large earthquakes. These data enable long-term hazard forecasts of the sizes and locations of earthquakes and estimates of the resulting ground motions [2]. Earthquake and aftershock forecasting can also provide the probabilities of earthquakes of certain magnitudes in a location over somewhat shorter time periods [[3], [4], [5]]. Other than these probabilistic forecasting approaches, specific warnings for shaking are only possible once an earthquake is underway [6]. Warning time is a crucial element in mitigating the impact of an earthquake on vulnerable communities. Perceiving and communicating this threat to communities likely to be impacted is a primary concern for emergency management and is what sparked the creation of earthquake early warning systems.

Earthquake early warning (EEW) systems function by utilizing a network of seismic sensors to monitor a region and rapidly detect seismic waves as they propagate away from the hypocenter [[6], [7], [8]]. Based on measurements made on the earliest seismic waves, EEW systems can determine the communities about to be impacted by shaking [7]. The amount of advance warning available is location and event dependent, with those farther from the epicenter receiving the most advance warning and those closest to the epicenter having little to no advance warning [9]; this is sometimes referred to as the “late alert zone” or “blind zone” [10]. Meier et al. [11] evaluated EEW system warning times in Japan and found that for locations that experienced moderate shaking (Modified Mercalli Intensities (MMI) IV-VI) most sites could receive at least 10 s advanced warning, with areas experiencing higher ground motions generally receiving shorter warning times. McGuire et al. [10] studied EEW system warning times in the United States (US) Pacific Northwest, which includes our study area of Seattle, Washington. Warning times for moderate and larger ground motions (MMI 4.5) were typically less than 20 s, but in extreme cases could be as much as 90 s. Note that, in the study, alerts were assumed to be distributed as soon as even low ground motions (MMI 2.5 or 3.5) were detected to increase available warning times, following the recommendations of Minson et al. [9]. These seconds hold the potential to change the way that individuals and communities respond to the earthquake. When coupled with automation, this advanced warning may be used by a wide variety of industrial applications to mitigate the earthquake's impact [12,13]. With sufficient warning time, EEW systems have the potential to change the protective actions available to individuals before and during the earthquake to mitigate impacts [14].

Different nations take a diverse array of approaches to recommended protective actions [15] and earthquake early warning [16]. While no single protective action fits every situation, the current recommended action for people inside a building during an earthquake can be separated into two primary options [16]. The first is to “drop, cover, and hold on” and the second is to evacuate the building, particularly if the structure is not built to withstand seismic impacts. For buildings that are built to adequate seismic-related building codes, the standing recommendation is to remain in the building and to “drop, cover, and hold on” [17]. The recommendation to drop, cover, and hold on is largely due to the risk of internal, often nonstructural, components falling and impacting people within the structure [18]. Evacuating a building may be recommended when the primary threat to occupants is the building itself collapsing, rather than internal objects falling [19]. Building collapse may occur if it is not adequately designed to resist earthquakes. EEW may not provide sufficiently long warning times to evacuate buildings, particularly multistory structures. In these scenarios, multiple responses may be recommended. An example is in Mexico City where different floors of structures are given different instructions [20]. The lower floors are instructed to use the time given by the EEW system to evacuate the structure, whereas the upper floors are instructed to find cover within the structure [21]. An EEW system may allow for a third protective action in which advance warning could enable individuals to move efficiently within a building to a more protected location, such as from a lecture hall to an adjacent study space with sturdy tables [21,22].

In this study, we use agent-based methods to simulate an individual responding to earthquake early warning messaging. We chose agent-based modeling (ABM) because it is effective at simulating real-world scenarios that involve autonomous interacting agents [23]. ABM has been extensively used to simulate pedestrian fire evacuations [24,25] and has been shown to be a powerful tool for applying simple rules to guide agent (people) interactions, both with other agents and with their environment [26]. There have also been preliminary studies where ABM was applied to earthquake and post-earthquake evacuations [[27], [28], [29]]. This study is the first to apply ABM to assess the impact of an EEW system on protective actions in an earthquake. While we use a single agent within an apartment environment as a proof of concept of the methodology, ABM is capable of scaling to simulate multiple, or even hundreds, of agents in a variety of environment types and settings [26,30,31]. The capacity of ABM type models to upscale to more complex simulations is a major strength in using this type of modeling when simulating the responses of people, both as individuals and as groups. [26,30,31]. Simulations can guide understanding of new tools, such as EEW systems, and provide some ‘field’ testing of these tools without needing an actual earthquake to occur. This supports refinement of the tools to potentially improve mitigation action [[30], [31], [32]].

EEW systems are relatively new, and many users do not know how to best use the warning they provide [33]. The present study applies agent-based modeling (ABM) to investigate how to optimize the protective actions taken by individuals during an earthquake when EEW is available. Agent-based simulation provides a tool for improving protective action recommendations to EEW system users. This study represents the first time agent-based simulations have been applied to EEW systems. This preliminary application of ABM to EEW systems shows that ABM has the potential to inform the protective actions recommended in response to EEW alerts.

## 2 Methods

When building agent-based models it is important to represent the real world accurately so that the model can produce useful results [23]. The Unity game engine [34] was used to develop and run the presented simulation. This engine was selected because of its flexibility as a leading video game engine. It is designed to be customizable and incorporate multiple character decisions as well as complex environment conditions [35]. Similar software tools have been used in fire evacuation studies [[35], [36], [37]]. This game engine also allows for future upscaling of the simulation to incorporate more complex parameters and additional agents and agent types and for custom adjustments of environment and agent parameters to better represent the real-world system in question. The application of these parameters is detailed below. Two purchased assets were applied in the simulation. Agent pathfinding was based on the Unity asset *Astar Pathfinding Project* [38] and internal structures and furniture were added using the Unity asset *House Furniture Pack* [39].

### 2.1 Environment parameters

**Fig. 1:**
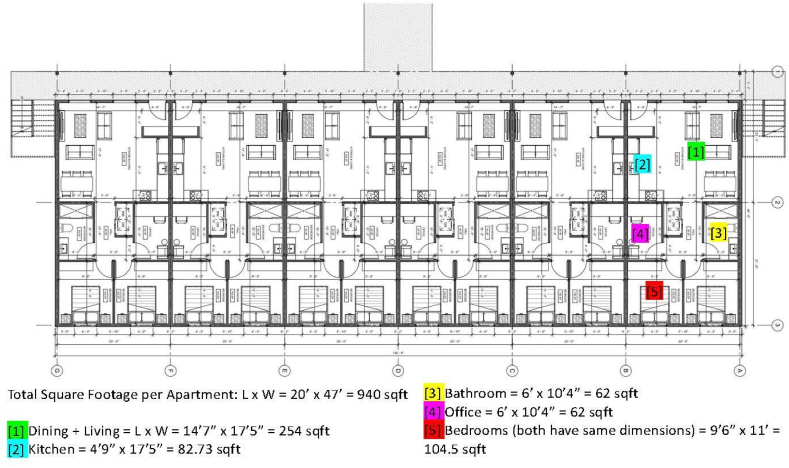
Annotated floorplan [41] of a representative residential-use URM building in the Seattle, Washington area. These structures are typically 3–4 stories tall. The simulation uses a ground floor apartment unit. Annotations are used for building the simulation to scale.

The environment for the ABM simulation was an apartment unit from an unreinforced masonry (URM) building in Seattle, WA. We selected Seattle, WA as the study area because of the potential for relatively long EEW warning times there [10], and its proximity to the Cascadia Subduction Zone and other seismic hazards in the Pacific Northwest [40]. Seattle has many URM buildings [41]. We focused on a residential use URM building because most of the deaths associated with earthquakes occur in and from such buildings [42], as URM buildings pose the greatest risk of collapse [43]. Further, Seattle's URM database [41] was helpful in selecting an apartment floor plan that was representative of similar buildings throughout the study area. We decided on a residential building because most video data is in residences. A residential use URM building in the Seattle area is typically three to four stories tall. The apartment unit, shown in Fig. 1 is located on the ground floor, with the egress point opening away from the building, and not into a corridor or other passageway [41].

**Fig. 2:**
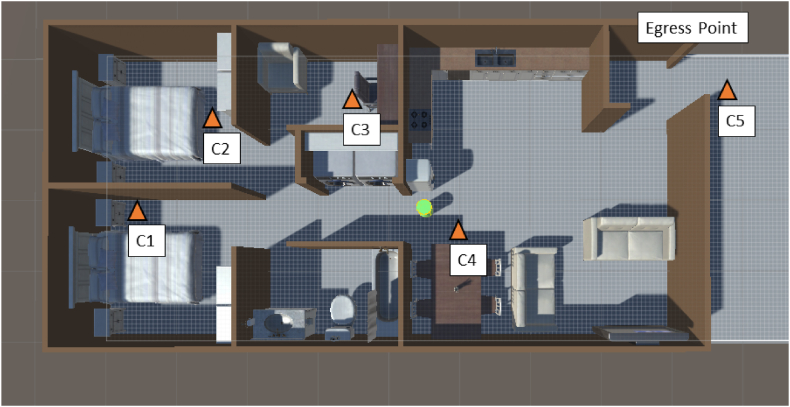
View of the simulation with cover locations marked. Cover locations are shown by orange triangles and labeled C1 to C5, with C5 representing evacuating the building. The egress point (front door) is also labeled. Actual cover locations will vary based on arrangement and furniture types. The agent is represented by the green cylinder in the middle.

The simulation was run with five potential cover or safe locations (see Fig. 2). Each of the four interior locations (C1-C4) is inside the apartment and assumes the presence of a sturdy furniture piece, such as a table or desk. Locations were placed in each of the two bedrooms, the office, and the dining area of the front living space. The fifth location (C5) represents evacuating the apartment and was located just outside of the egress point (i.e., front door).

### 2.2 Agent parameters

**Fig. 3:**
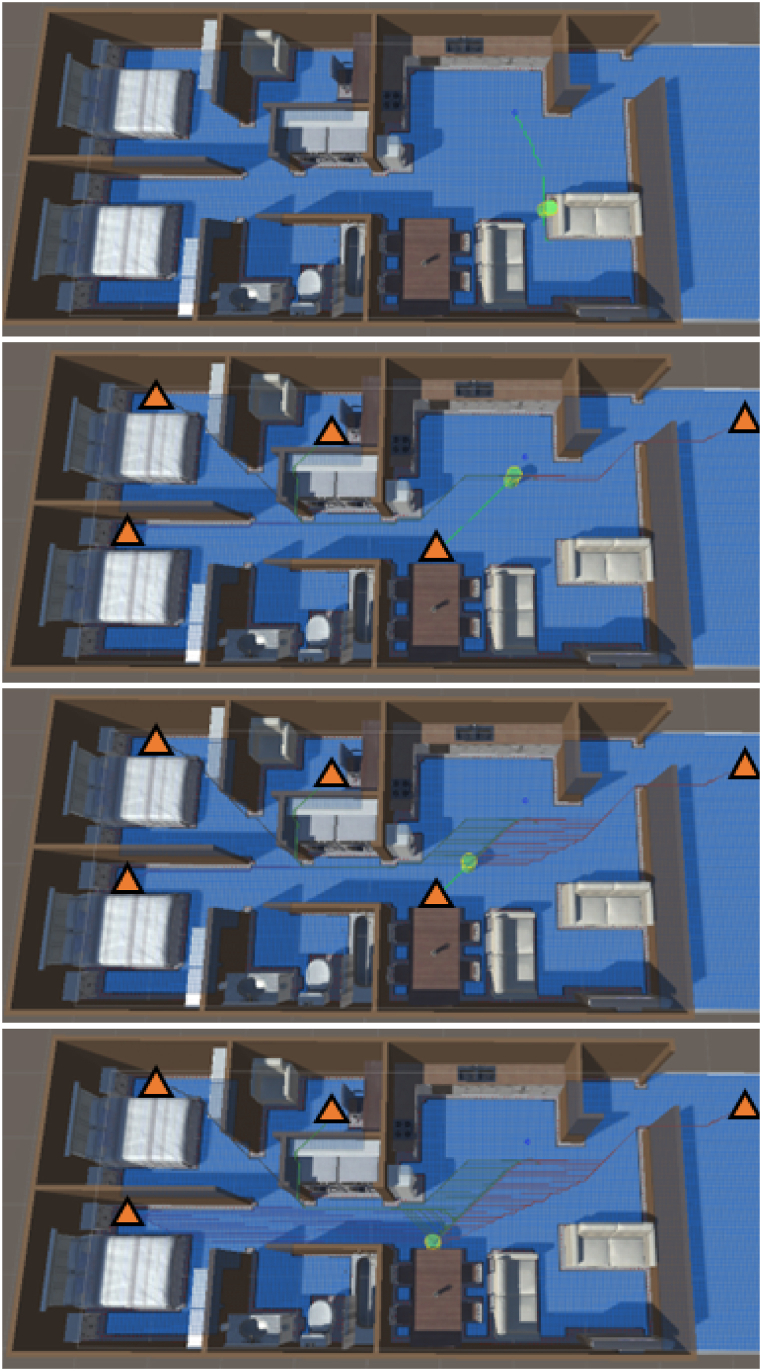
Snapshots from a simulation iteration. The agent is shown by the green cylinder in all panels. The top panel shows the agent in the initial random walk. The green arrow shows the direction of movement, and the green highlighted path indicates the path the agent will traverse. In this iteration the agent responds to the EEW message just before reaching the random walk destination. First, the agent pauses for 2–6 s (randomly selected in each iteration) to simulate cognitive processing and then begins to act on the warning message. The agent maps a path to each of the five cover locations (shown by the orange triangles in the lower three panels). The second panel shows the change in movement direction. The paths to each of the cover locations are also shown. The agent selects the shortest of these paths and moves to the corresponding cover location. The agent stops moving once it reaches the nearest cover location. In this case it was the dining area table. At this point the simulation stops and records the time from when the agent received the warning message to when it reached a cover location. While the present simulation does not simulate this, it is assumed that individuals drop, cover, and hold on upon reaching a place of cover. Future work on this or similar simulations may benefit by adding this feature as well.

**Fig. 4:**
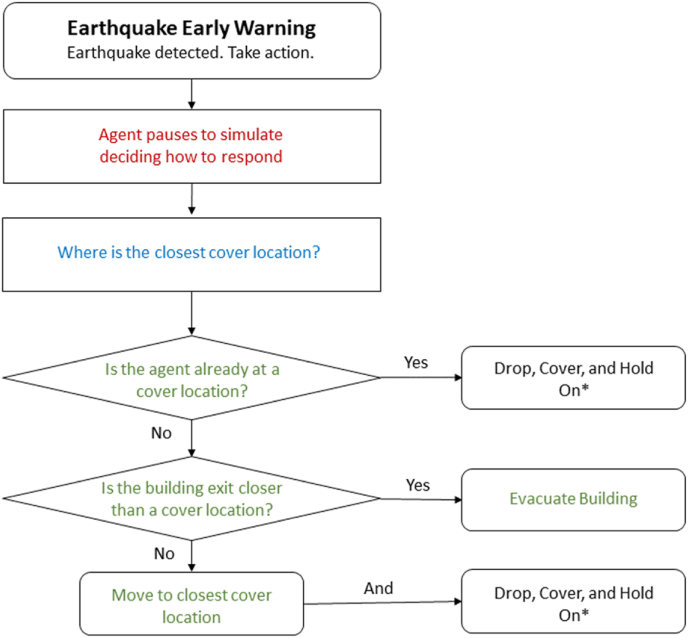
Shows cognitive framework of the agent. The ∗ next to ‘drop, cover, and hold on’ indicates that the agent does not actually perform this action in the present simulation. At this stage the agent stops moving upon reaching the cover location. Future work on this or similar simulations may benefit by including the drop, cover, hold on actions. The colors are correlated with the colors used in Algorithm 1.

The simulation begins with the agent moving in a random walk within the apartment unit (see Fig. 3, Fig. 4). After a random time between five and 10 s (labeled as ‘random walk timer’ in Fig. 4 and Algorithm 1), the agent is given an EEW message. The random walk time is to allow the agent to be in a randomized location within the apartment when triggered to move to a cover location. Upon receiving the EEW message, the agent pauses between two and 6 s before then moving towards the nearest cover location (labeled as ‘pause time’ in Fig. 4 and Algorithm 1). The pause time applied here represents the time needed for an individual to process the warning message and then begin to take protective action. The time interval was determined by annotating the timing of protective actions taken by people experiencing earthquake shaking in video footage of the Anchorage 2018 7.1 magnitude earthquake, gathered from public-access social media [44]. Two and 6 s were, respectively, the minimum and maximum time that people took to begin responding to ground shaking in the video footage. Of course, ground shaking is likely to be a stronger trigger for taking protective action than an EEW warning message. However, since there is little-to-no video footage available for observing the response of people responding to an EEW message, this is used as a best-case proxy in this simulation.

After the pause interval the agent then plots the shortest route to each of the five cover locations (C1 to C5 in Fig. 2). After selecting the shortest of the possible paths, the agent moves along the calculated path to the cover location. When the agent reaches the cover location it stops moving and a timestamp is recorded of the time from the beginning of the pause interval (when the agent received the EEW message) to when it reached a cover location (labeled as ‘protective action timer’ in Fig. 4 and Algorithm 1). Fig. 4 presents a cognitive framework of the agent's steps and decisions as well as a pseudocode example of the algorithm used to direct the agent in the simulation.

Algorithm 1

**Figure:**
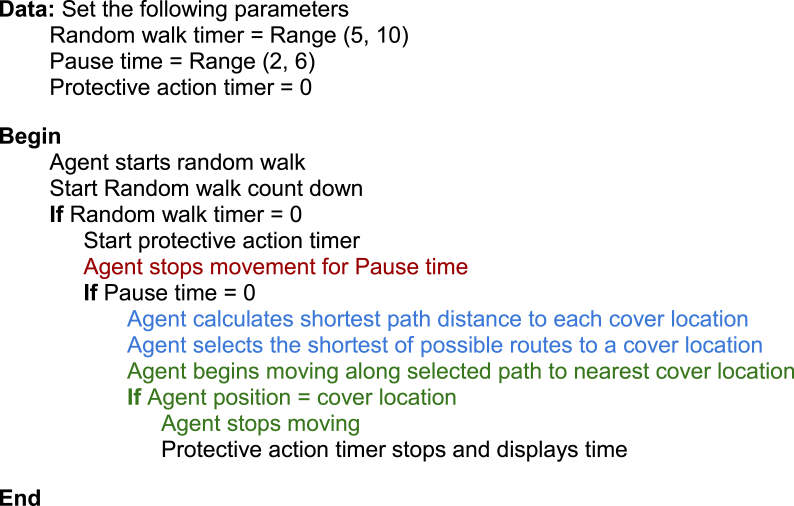
Pseudocode for how the agent's pathfinding simulates the cognitive framework. Colors indicate correlated functions between the cognitive framework (Fig. 4) and the algorithm.

**Fig. 5:**
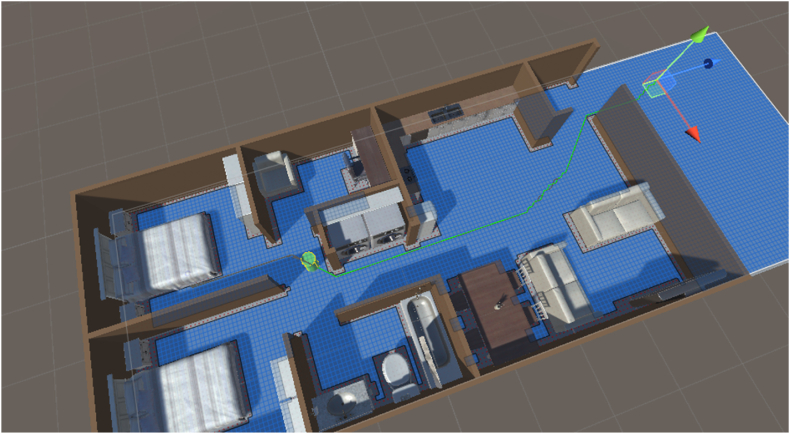
View of the simulation where evacuation was the only allowed protective action. The agent is represented by the green cylinder and the path to the evacuation point is shown by the green line exiting the apartment. The evacuation point (C5 from Fig. 2) is indicated by the directional curser. This parameter on average took the agent longer to traverse and distances covered were greater.

Initially all five cover locations (C1 to C5 in Fig. 2) were available to the agent and there was no priority given towards evacuating or moving to cover within the apartment unit. The agent determined which action was best by which cover location was closest when the EEW message was received. In cases where the structure is known to have a relatively high risk of collapse, it may be advantageous to place higher priority on evacuation. To simulate giving evacuation a higher priority, and to compare timing results to using internal cover locations, the simulation was adapted so that the only protective action available to the agent when it received the EEW message was to evacuate the apartment unit. Fig. 5 illustrates an example where the agent was allowed to traverse only to the exterior cover location (C5).

The movement speed of the agent has a significant impact on how quickly it is able to reach the various cover locations in the simulation. Likewise, the movement speed and capabilities of individuals varies widely and directly impacts their ability to take protective actions. The simulation was run using two movement speeds. These movement speeds were selected based on the walk speeds used in traffic modeling in the US. The current walk speed used in traffic models is 3 ft/s (0.91 m/s). Prior to January 2006, the walk speed was 4 ft/s (1.2 m/s) [45]. Montufar et al. [45] investigated the impact that age and gender had on the normal and crossing walk speeds of pedestrians. They found that a walk speed of 4 ft/s was above the average normal walk speed of older (65 years and older) pedestrians and that a walk speed of 3 ft/s better represented this age category in traffic models. Since there is little to no video footage of people responding to EEW messages, the simulation was run using both the 3 ft/s and 4 ft/s walk speeds that have been the standard in traffic models.

## 3 Results

**Fig. 6:**
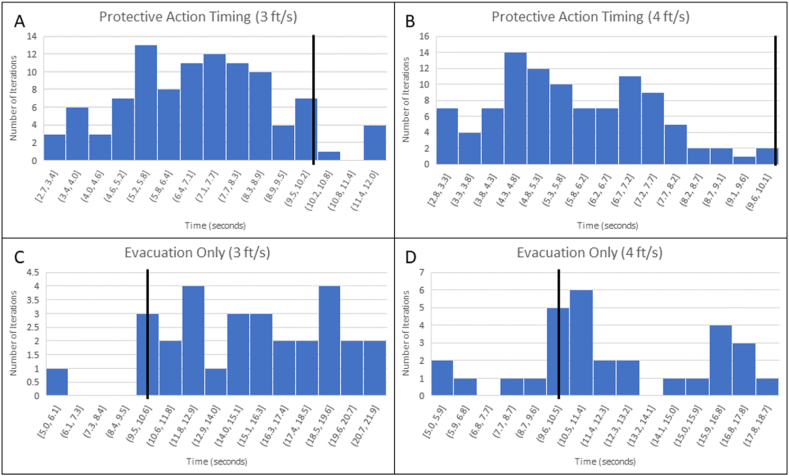
Histograms showing the distribution of the time taken by the agent from receiving the EEW message to reaching a cover location. For all histograms the black line indicates Meier et al.’s [11] results that most sites with moderate ground shaking (MMI ∼ 4–6) had at least 10 s warning time. Panels A and B show the distributions for agent movement speeds of 3 ft/s (0.91 m/s) and 4 ft/s (1.2 m/s), respectively, when all cover locations (shown in Fig. 2) were available to the agent. Panels C and D show the distributions for movement speeds of 3 ft/s (0.91 m/s) and 4 ft/s (1.2 m/s), respectively, when evacuation was the only available option for the agent. Panels A and B indicate that for a single agent in an apartment unit setting it is reasonable for them to reach cover before significant ground shaking begins at their location. Panels C and D indicate that it is more difficult to evacuate a structure prior to the onset of ground shaking even with the advance warning time provided by EEW systems. In both protective action responses increasing the movement speed of the agent decreased the time needed to complete the protective action. For Panels A and B, N = 100; for Panels C and D, N = 30 [46].

When all potential cover locations (C1 to C5 in Fig. 2) were available to the agent, and with a movement speed of 3 ft/s, the average time to respond to an EEW message and move to a cover location was 6.96 s. The minimum time was 2.74 s, and the maximum was 12 s (Fig. 6A and Table 1). With a movement speed of 4 ft/s, the average time was 5.81 s, the minimum time was 2.84 s, and the maximum time was 10.12 s (Fig. 6B and Table 1). Outside of the late-alert zone, EEW systems are typically able to provide warning times ranging from a few seconds to a few tens of seconds for moderate to strong ground motions. Longer warning is possible at sites far from large magnitude earthquakes where ground motions are less likely to result in damage [10,11]. Given these time ranges, the simulation results suggest that EEW systems are capable of providing sufficient warning time for an individual to reach cover within an apartment setting prior to major ground shaking (Fig. 6).

**Table 1:** Table showing the difference in the time to reach cover for the agent under both movement speeds with all cover options available to the agent.

All Cover Options			
	Time-to-Cover (seconds)		
Movement Speed	Average	Min	Max
3 ft/s (0.9 m/s)	6.96	2.74	12.01
4 ft/s (1.2 m/s)	5.81	2.84	10.12

It is important to note that, in this simulation, the agent is aware of the potential cover locations in the area and its proximity to them. In real world situations this result would only be possible to replicate if the individual was similarly aware of the cover locations in their surroundings. This supports the concept that an individual's knowledge of their surroundings is of vital importance to their ability to effectively take protective action upon receiving an EEW message [47,48].

Evacuation was also one of the possible protective actions in this simulation. Simulation iterations where evacuation (C5, Fig. 2) was the only allowed protective action, with a movement speed of 3 ft/s, had an average evacuation time of 15.35 s, with a minimum of 5.01 s and a maximum of 21.87 s (Fig. 6C and Table 2). With a movement speed of 4 ft/s, the average evacuation time was 12.26 s, with a minimum time of 5.01 s and a maximum time of 18.67 s (Fig. 6D and Table 2). For both movement speeds, this was approximately double the time compared to when the agent had access to cover locations C1 through C5. The longer time required to evacuate the building in the simulation pushes closer to the maximum time ranges typically given by EEW systems [11].

**Table 2:** Table showing the difference in the time to reach cover for the agent under both movement speeds with only the evacuation option available to the agent.

Evacuation Only			
	Time-to-Cover (seconds)		
Movement Speed	Average	Min	Max
3 ft/s (0.9 m/s)	15.35	5.01	21.87
4 ft/s (1.2 m/s)	12.26	5.01	18.67

## 4 Discussion

Comparing the time required to reach internal cover locations (C1 to C4) with the time required to evacuate (C5) indicates that evacuation typically requires more time to complete, as expected. Minimizing the time to reach cover is critical because Shoaf et al. [49] found people who moved around during shaking were twice as likely to be injured than those who were not moving. It is also important to note that the results of this simulation present essentially a best-case scenario for evacuation. As shown in Fig. 2, the egress point for the apartment is relatively close because the interior space is small compared to other structures. In addition, this simulation has the egress point opening to an unrestricted, open area that is assumed to be safe and doesn't require traversing to a different floor. This is often not the case for other structures where the egress point may lead into an interior hallway. In addition to typically requiring more time than moving to internal cover locations, evacuating a structure may expose individuals to additional hazards, such as falling debris from non-structural components including brickwork or breaking glass [50]. Of course, not evacuating may expose individuals to structural collapse, the risk of which depends on the type of structure and the intensity of shaking. This emphasizes the importance of public outreach regarding what people need to know, and do, in the event of receiving an EEW message. Adding instructional content to the EEW system app could help individuals determine what is best for their individual situation and surroundings.

While this simulation allows for two possible protective actions—moving to an interior cover location or evacuating the structure—the actual range of observed behaviors in earthquake responses are much more diverse [44]. Behaviors range from effective protective actions including drop, cover, and hold on, to less effective behaviors such as sheltering under door frames. Individuals also engaged in actions that are not protective actions at all but are rather social responses, such as gaining proximity to children, alerting others, or using mobile devices to record events [44]. Effectively simulating this wide range of behaviors is valuable but complex. At this stage, we only investigate the potential for EEW messages to allow individuals to achieve cover prior to intense ground shaking at their location. In addition, as EEW systems become more familiar and trusted by the public, individuals’ responses may become more streamlined towards the protective actions of locate cover then, drop, cover, and hold on, or potentially evacuating unstable structures.

Due to limited data of people's responses to active EEW systems during an earthquake it is currently difficult to validate the simulation. Validation, and further improvement, of this and more complex simulations would benefit from the availability of data showing how people respond to receiving an EEW message. Video footage, similar to what was used to guide this simulation, showing individuals receiving an EEW message, and then reacting to the advance warning, would be ideal for comparing the results of the simulation to real-world use of EEW messaging. As EEW systems continue to be utilized, this data will become more available and allow for more accurate and complex simulations of how EEW messaging may improve people's ability to take protective actions during an earthquake.

Currently, the impact of EEW on populations is understudied. This is in part due to the relatively recent development of EEW systems and to the low number of counties with active EEW systems [21]. Multiple studies have been conducted that simulate people's responses during an earthquake [[51], [52], [53], [54]]. While such simulations are valuable for earthquake safety, they do not provide an effective comparison to the simulation used in this study as they focus on how people respond to earthquake induced ground shaking and this study focuses on how people may use EEW messages to respond prior to feeling earthquake induced ground shaking.

Fire represents an area of hazard research that incorporates early warning systems. Fire evacuation literature has much to offer in terms of protective actions and evacuation techniques, protocols, and procedures for that hazard. However, as we reviewed the fire evacuation literature, it became clear that there were several issues that made fire evacuation literature not as useful as we had hoped. One is the amount of time for evacuations. Most EEW systems—and specifically ShakeAlert, the EEW system for the West Coast of the United States we based our model on—offer less than 10 s of notice [55]. Evacuees from buildings require much more time than 10 s to evacuate successfully from most structures and situations, with Lin and Wu [56] suggesting that people need 4.8 min median time to evacuate a building fully in the case of fires. Second, fire provides other physical warnings like smoke and heat to alert people that a fire is occurring [57]. As EEW requires technical systems to detect earthquake induced seismic waves prior to shaking; any physical warnings like shaking and sound will provide similar amounts of timing [16].

This simulation is the first time that agent-based simulation has been applied to EEW systems. As such, there are multiple ways that the present simulation could be expanded upon to further improve its usefulness in guiding protective actions during earthquakes. One option is adding multiple agents to the simulation. ABM is particularly effective at simulating and observing emergent phenomena [26,30,31]. Emergent phenomena can occur when agents interact with each other and are unlikely to be represented with a single agent. Possible emergent phenomena include congestion in a hallway as multiple agents attempt to traverse at the same time or multiple agents attempting to use the same cover location(s). ABM's application of simple agent instructions creates an environment to observe how these agents interact and impact each other as they carry out their individual behavior.

Introducing multiple agent types would be another valuable expansion to the present simulation. This would allow for simulation of parent-child and caregiver interactions and responses to EEW messages. Incorporating multiple agent types would allow for greater range in agent mobility and could begin to simulate various ages, physical abilities, and the impacts of psychological factors in responding to EEW. Simulating the impact of leaders, such as a teacher in a classroom, on a group could also be valuable in guiding protective actions or training for future earthquake response [58].

ABM also allows for the expansion of the simulation environment. Possible ways to expand the present environment include adding additional apartment units to the simulation. This would be especially helpful in simulating emergent phenomena occurring during evacuations. Each apartment unit will be similar to the present simulation; however, the space outside of the apartment units may grow congested quickly if multiple agents rapidly evacuate the building. Adding multiple stories to the structure or adding interior complexity such as stairs may be helpful in guiding protective actions for additional scenarios. ABM is also capable of simulating hundreds to thousands of agents [30,31] and may be used to simulate responses in larger structures such as schools or hospitals.

Additional benefits that may come through continued use of simulations of EEW responses include helping emergency managers simulate cascading hazard scenarios that involve earthquakes. Such scenarios may highlight protective actions that have an impact on later hazardous events that people may encounter following the earthquake and associated ground shaking. Possible cascading events associated with earthquake ground shaking include entrapment within damaged structures, potential small- and large-scale HAZMAT releases, possible tsunami inundation, and transportation interruptions. Cascading hazards and disasters are greatly impacted by the social vulnerabilities in a community [[59], [60], [61], [62]]. Scenario generation, and simulation of cascading events, may help alleviate some of these vulnerabilities before they result in disasters [32]. Whether combined with possible cascading events or left at responding to EEW alerts, simulations of protective actions may provide a useful public outreach tool for educating communities about potential protective actions. Use of simulations may increase public awareness of earthquake hazards (immediate and cascading), and improve the public's understanding of EEW systems and how they may be used to improve protective actions taken in the event of an earthquake.

## 5 Conclusion

EEW systems are a powerful addition to our ability to respond to earthquakes. The advance warning these systems provide allows for more effective application of protective actions. This initial application of ABM to EEW systems shows that the warning time provided by EEW systems can allow an agent to reach cover before severe ground shaking occurs at their location. We also found that there is generally insufficient time for the agent to evacuate the structure under the examined parameters. Providing people with appropriate recommendations of protective actions that are achievable (or not) in the available warning time has the potential to reduce the severity of injuries experienced during an earthquake. Augmenting this work with additional simulations of responses to EEW systems could provide information on how to prepare people and communities to respond to hazardous conditions created by earthquake induced ground shaking.

## CRediT authorship contribution statement

**Matthew Wood:** Writing – original draft, Visualization, Validation, Software, Methodology, Formal analysis, Conceptualization. **Sara K. McBride:** Writing – review & editing, Supervision, Methodology, Funding acquisition, Conceptualization. **Xilei Zhao:** Writing – review & editing, Supervision, Funding acquisition, Formal analysis, Conceptualization. **Dare Baldwin:** Writing – review & editing, Methodology. **Elizabeth S. Cochran:** Writing – review & editing, Formal analysis. **Xiaojian Zhang:** Writing – review & editing, Methodology, Conceptualization. **Nicolas Luco:** Writing – review & editing, Methodology. **Ruggiero Lovreglio:** Writing – review & editing, Software, Methodology. **Tom Cova:** Writing – review & editing, Supervision, Resources, Methodology, Funding acquisition, Formal analysis, Conceptualization.

## Data availability statement

Data for the agent-based simulation iterations has been added to a publicly available repository [46].

## Funding statement

The study was funded by the United States 10.13039/100000001National Science Foundation, grant #1921157.

## Declaration of competing interest

The authors declare that they have no known competing financial interests or personal relationships that could have appeared to influence the work reported in this paper.
